# Comparison of the biomechanical differences in the occlusal movement of wild-type and BMP9 knockout mice with apical periodontitis

**DOI:** 10.3389/fbioe.2022.1036061

**Published:** 2022-10-17

**Authors:** Mengtian Peng, Xiaofei Ma, Yuying Cao, Chunjuan Wang, Qin Tan, Xinyue Chu, Pengfei Zhou, Tiwei Fu, Panpan Liang, Shidian Ran, Tong-Chuan He, Jinhua Wang, Enyi Huang

**Affiliations:** ^1^ Chongqing Key Laboratory of Oral Diseases and Biomedical Sciences, Stomatological Hospital of Chongqing Medical University, Chongqing, China; ^2^ Chongqing Municipal Key Laboratory of Oral Biomedical Engineering of Higher Education College of Stomatology, College of Stomatology, Chongqing Medical University, Chongqing, China; ^3^ Department of Stomatology, Children’s Hospital of Chongqing Medical University, Chongqing, China; ^4^ Molecular Oncology Laboratory, Department of Orthopaedic Surgery and Rehabilitation Medicine, The University of Chicago Medical Center, Chicago, IL, United States

**Keywords:** apical periodontitis, occlusal movement, BMP9, biomechanics, finite element analysis

## Abstract

Apical periodontitis is a common clinical disease caused by bacteria; bacterial metabolites can cause an imbalance in bone homeostasis, bone mass reduction, and tooth loss. Bone resorption in apical periodontitis causes a concentration of stress in the tooth and periodontal tissues during occlusion, which aggravates the disease. Emerging evidence indicates that bone morphogenetic protein 9 (BMP9), also known as growth differentiation factor 2(Gdf2), may play an important role in tooth and dentoalveolar development. Herein, we investigated the role of BMP9 in the development of apical periodontitis and its effects on the biomechanics of dentoalveolar bone. Apical periodontitis models were established in five BMP9 knockout (KO) mice and five C57BL/6 WT (wild-type) mice. At baseline and 14, 28, and 42 days after modeling, *in vivo* micro-computed tomography analysis and three-dimensional (3D) reconstruction were performed to evaluate the apical lesion in each mouse, and confirm that the animal models were successfully established. Finite element analysis (FEA) was performed to study the stress and strain at the alveolar fossa of each mouse under the same vertical and lateral stress. FEA revealed that the stress and strain at the alveolar fossa of each mouse gradually concentrated on the tooth cervix. The stress and strain at the tooth cervix gradually increased with time but were decreased at day 42. Under the same lingual loading, the maximum differences of the stress and strain at the tooth root in KO mice were greater than those in WT mice. Thus, these findings demonstrate that BMP9 could affect the biomechanical response of the alveolar fossa at the tooth root in mice with apical periodontitis. Moreover, the effects of BMP9 on the biomechanical response of the alveolar bone may be site-dependent. Overall, this work contributes to an improved understanding of the pathogenesis of apical periodontitis and may inform the development of new treatment strategies for apical periodontitis.

## Introduction

Apical periodontitis is an inflammatory disease that occurs in the tissues around the tooth root, and this disease progresses slowly and has a long course. The pathogen underlying this disease mainly comes from the dental pulp. When dental pulp disease causes persistent infection, bacteria and their metabolites can spread to the tissues around the root through the apical foramen, leading to chronic apical periodontitis (Nair, 2006; Braz-Silva et al., 2019). Since inflammation not only promotes osteoclast differentiation but also inhibits osteoblast function, chronic apical periodontitis often involves adjacent dentoalveolar bone and cementum at the root tip, resulting in bone resorption and tissue destruction at different sites (Alvarez et al., 2019). An understanding of the pathological process underlying apical periodontitis is crucial for developing effective treatment strategies. Mechanical factors and an imbalance between osteogenesis and osteolysis are important factors affecting the pathological process of apical periodontitis. The loss of bone tissue that occurs in apical periodontitis leads to stress concentration in the tooth and periodontal tissues during occlusion. The stress in the tooth increases with the loss of bone tissue, which aggravates the disease (Dal Piva et al., 2017).

Numerous signaling pathways have been implicated in the regulation of bone metabolism, including TGF-β/BMP superfamily members, WNT/β-catenin signaling, and NOTCH ligands and receptors (Zhao et al., 2021). Among these regulators, BMPs represent a group of the most potent osteogenic factors. The BMP signaling pathway plays a crucial role in bone mass maintenance by regulating the differentiation and anabolic bone-formation activity of osteoblasts and osteocytes (Phimphilai et al., 2006; Zhu et al., 2022). BMP9 (also known as growth and differentiation factor 2, or GDF2) is one of the least characterized yet most potent osteogenic BMPs (Luu et al., 2007; Luther et al., 2011). Recent studies have shown that BMP9 may play an important role in tooth and dentoalveolar development. BMP9 can regulate dentin formation, root development, and alveolar crest height (Saulacic et al., 2018; Li et al., 2021a). BMP9 knockout (KO) mice show significant defects in the dentoalveolar bone complex (Huang et al., 2019). Interestingly, a recent study also found that BMP9 can protect cells in the inflammatory state (Li et al., 2021b).

Finite element analysis (FEA) can be used to establish comparable, visual, and standardized three-dimensional (3D) models and is one of the most common methods to study stress values and distributions. According to this method, the whole structure is regarded as finite elements connected to one another, and the mechanical properties of the whole structure can be understood by studying the characteristics of each element in turn and combining the mechanical properties of each element. Due to its high feasibility, high accuracy, and low cost, FEA has been widely used as a mechanical analysis tool for the study of oral biomechanics (Mangado et al., 2016; Wang et al., 2022). In recent years, an increasing number of studies have introduced 3D FEA into root canal therapy (Jang et al., 2014; Gomes É et al., 2015; Askerbeyli Örs et al., 2019). However, the biomechanics of the dentoalveolar bone in the pathological process of apical periodontitis remain to be established.

Therefore, this study investigated the role of BMP9 and dentoalveolar bone biomechanics in the development of apical periodontitis. A model of apical periodontitis of the left mandibular first molar was established in BMP9 KO mice and wild-type (WT) mice. The stress and strain in the alveolar fossa of the left mandibular first molar at 0-, 14-, 28-, and 42-day following modeling were studied using FEA. Further, the distributions of stress and strain in the alveolar fossa of the mandibular first molars in KO and WT mice with apical periodontitis were compared.

## Materials and methods

### Experimental animals

**FIGURE 1:**
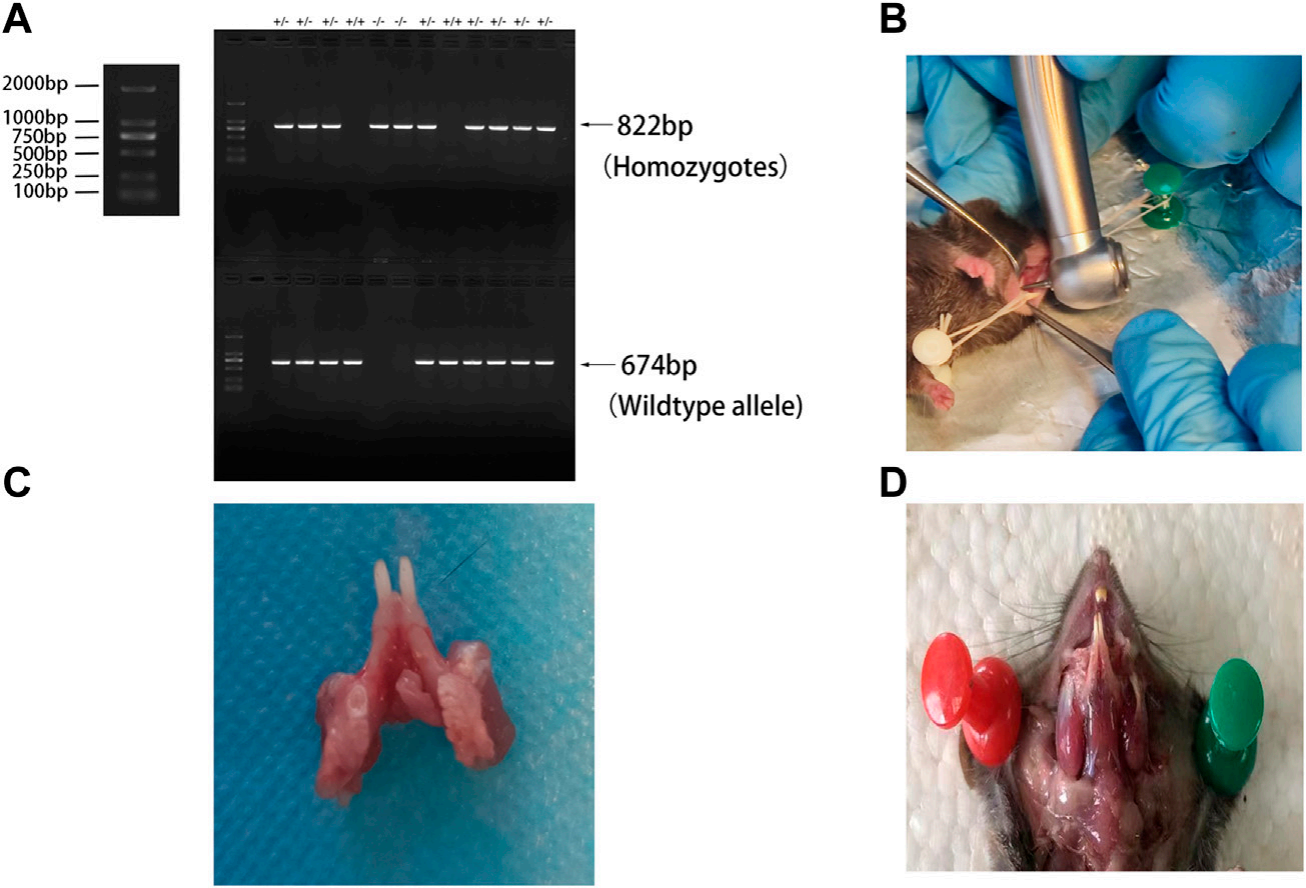
**(A)** Genetic identification; **(B)** periapical surgery modalities; **(C)** left mandibular first molar after modeling of apical periodontitis; and **(D)** masticatory muscle site in mice.

BMP9^+/−^crosses were purchased from Cyagen Model Organisms Center, Inc. (Suzhou, China). To obtain BMP9^−/−^ mice, female and male BMP9^+/−^crosses were intercrossed randomly. Offspring mouse genotypes were identified by PCR using two pairs of primers: F1 (5′-CAA​GGC​TGG​TCA​ATA​TGC​ATC​AGA​TA-3′) and R1 (5′-AGA​TGG​TCA​CCG​AAT​CAA​TAA​AGG-3′); F2 (5′-GGG​TTA​GTG​GGT​AGA​AAG​ATG​TCA-3′) and R2 (5′-AGA​TGG​TCA​CCG​AAT​CAA​TAA​AGG-3′). The two primer pairs generated fragments of 822 bp and 674 bp from the BMP9 KO and wild-type alleles. The gene identification results are shown in Figure 1A. A total of five BMP9 KO(BMP9^−/−^) mice and five WT mice aged eight weeks were randomly selected for this study; each mouse weighed 20 ± 1 g. All mice were fed in the animal breeding room of Chongqing Key Laboratory of Oral Diseases and Biomedical Sciences. The room temperature was maintained at 25 ± 1°C, the relative humidity was 55 ± 5%, and the animals were housed under a 12-h light/dark cycle. This study was approved by the Ethics Committee of Chongqing Medical University.

### Establishment of the apical periodontitis model

A classical mouse model of apical periodontitis was established (Goldman et al., 2019). Each mouse was anaesthetized and immobilized on a foam board wrapped with tin foil (Figure 1B). The pulp of the mandible left first molar was exposed by a high-speed 1/4 round bur on a portable dental unit (Figure 1C) and probed with a size 8 endodontic file. The root canals were left open to the oral environment for spontaneous infection throughout the experiment. *In vivo* micro-computed tomography (CT) scanning was performed on days 0, 14, and 28 after modeling. The mice were sacrificed on day 42 after modeling. The mandibles were dissected, separated, and fixed in 4% paraformaldehyde.

### Micro-computed tomography scanning

On days 0, 14, and 28 after modeling, the mice were anesthetized and fixed in the prone position in a scanning tube. On day 42, the dissected specimens were placed in the center of a cylindrical piece of foam of a suitable diameter and were fixed in this special scanning tube. The scanning parameters were as follows: tube current 113 μA, tube voltage 70 KV. The resolution of *in vivo* micro-CT is consistent with that of *in vitro* micro-CT. The voxel spacing of micro-CT images was all 0.015 mm^3^ × 0.015 mm^3^ × 0.015 mm^3^.

### Analysis of apical lesion size

Analysis of apical lesion size was performed on the coronal surface of the mandibular first molar. The size of the apical lesion in the apical third of the mesial root canal was measured using ImageJ software; the values measured in square millimeters were compared between the KO and WT groups. The area of the periodontium in the proximal middle apical third of each mouse before modeling was measured. The area of inflammation at different time points was calculated by using the area of the apical lesion at different time points minus the area of the periodontal membrane before modeling (Siddiqui et al., 2019).

### Finite element model of the mandible

The micro-CT data were saved in DICOM (Digital Imaging and Communications in Medicine) format. Using the import command in Mimics 3D medical reconstruction software (Materialize), the CT images saved in DICOM format were imported to adjust the threshold values of the images. The 3D computing function was used to build original 3D models of each mandible, which were saved in STL format. The original STL models were reconstructed into editable UV-smooth-surface models using MAYA software in order to distinguish the cortical bone, cancellous bone, tooth, blood vessels, and nerves. MAYA software can simplify the number of faces and enables fast-batch Boolean operations. Then, the files were saved in “.mb” format and changed to “.stp”format. The models were imported into the 3D FEA software ANSYS and the meshes were divided automatically. Young’s modulus and Poisson’s ratio values were adopted from the relevant literature (Table 1) (Mankani et al., 2006; Buezas et al., 2019).

**TABLE 1:** Material properties.

Material	Young’s modulus (Pa)	Poisson’s ratio
Mouse cortical bone	2.50E + 10	0.3
Mouse tooth	3.00E + 10	0.3

### Loading and boundary conditions

**FIGURE 2:**
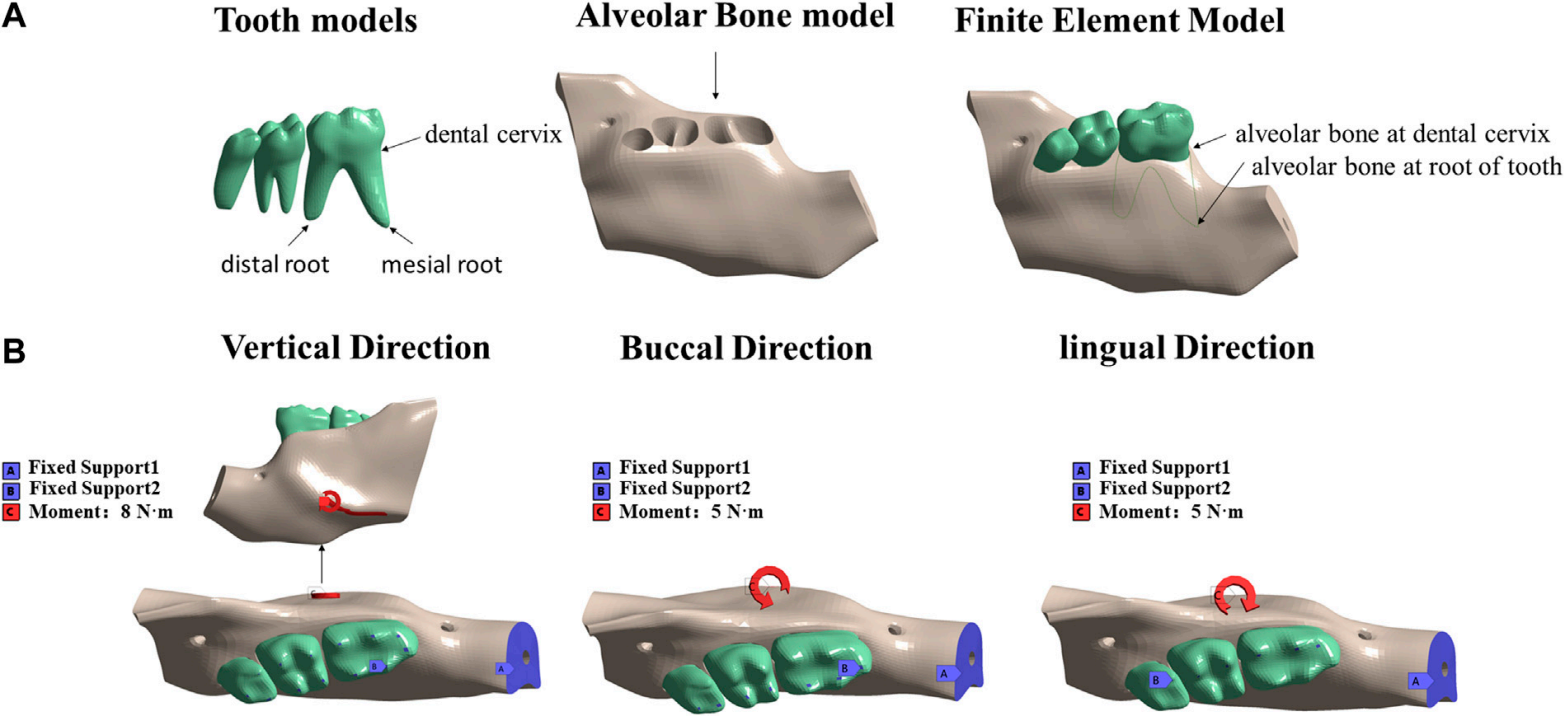
Resulting solid three-dimensional finite element model obtained in ANSYS: **(A)** tooth model, alveolar bone model, and intact model; **(B)** three force directions; fixed and stressed parts.

The model of mandibular movement during occlusal contact is shown in Figure 2A. The mastication activity of the jaw is a multidirectional movement. In this study, the masticatory muscle vectors acting on the mandible were simplified and analyzed in three different conditions: vertical occlusion, buccal occlusion, and lingual occlusion. The incisor zone of the mandible was fully restrained. Displacement in occlusal contact in the vertical direction was constrained over the cusp of the left mandibular molar. Buccal occlusion contact was restricted to the buccal inclined surface of the lingual cusp and lingual occlusion contact was restricted to the lingual inclined surface of the buccal cusp.

A load of 8 N·m was applied to the mandible from the vertical direction to simulate mandibular torsion in the vertical direction. A load of 5 N·m was applied horizontally to the mandible from the buccal and lingual directions to simulate the mandible twisting in the buccal and lingual directions (Figure 2B). All loads were applied to the external oblique line of the mandible, originating from the masticatory muscle attachment site in mice (Figure 1D). The stress and strain data of the alveolar bone of the mandibular first molar under the action of different forces were extracted.

### Hematoxylin and eosin staining

The mandibles of WT and KO mice were fixed in neutral paraformaldehyde solution (Servicebio, Wuhan, China) for 48 h, decalcified with 10% ethylenediaminetetraacetic acid (EDTA) (Servicebio, Wuhan, China), dehydrated through graded alcohol, embedded in paraffin, and then made into continuous sections. Paraffin slides were dewaxed, rehydrated, and subjected to H&E (Servicebio, Wuhan, China) staining. The sections were dehydrated through graded alcohol and immersed in xylene until transparent, sealing with neutral resin.

### Tartrate-resistant acid phosphatase staining

Paraffin slides were dewaxed, rehydrated, and incubated using TRAP incubation solution (acid phosphatase kit; Servicebio, Wuhan, China). Next, the slides were stained with hematoxylin and turned blue with ammonia. The slides were dehydrated with graded alcohol and immersed in xylene until transparent, sealed with neutral resin. The sections were observed using an optical microscope.

### Statistical analysis

All statistical analyses were performed using IBM SPSS 23.0 software. The quantitative measures were first tested for normality; those that were non-normally distributed were expressed using the median. The Mann-Whitney *U*-test was used for between-group comparisons of the quantitative measures that were not normally distributed. The significance level was set at 5%.

## Results

### Apical lesion area at different time points

**FIGURE 3:**
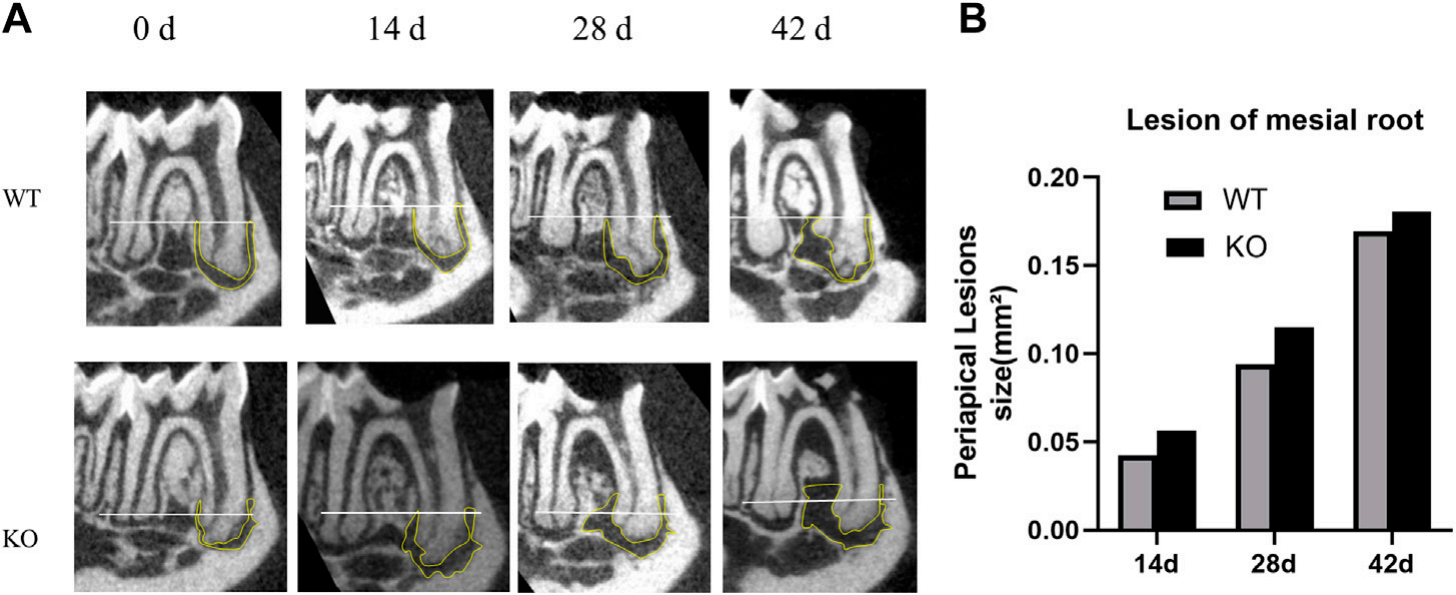
Micro-computed tomography analysis of periapical lesions after root canal treatment in mice. **(A)** Representative images of tooth in the KO group and WT group. White line passing through the apical third of the mesial root canal is denoted as the coronal limit of the periapical lesion. **(B)** Comparison of the differences in the size of periapical lesions in the mesial canal (*p > 0.05 indicates no significant difference; Mann–Whitney test). Data represent the median of each group.

**FIGURE 4:**
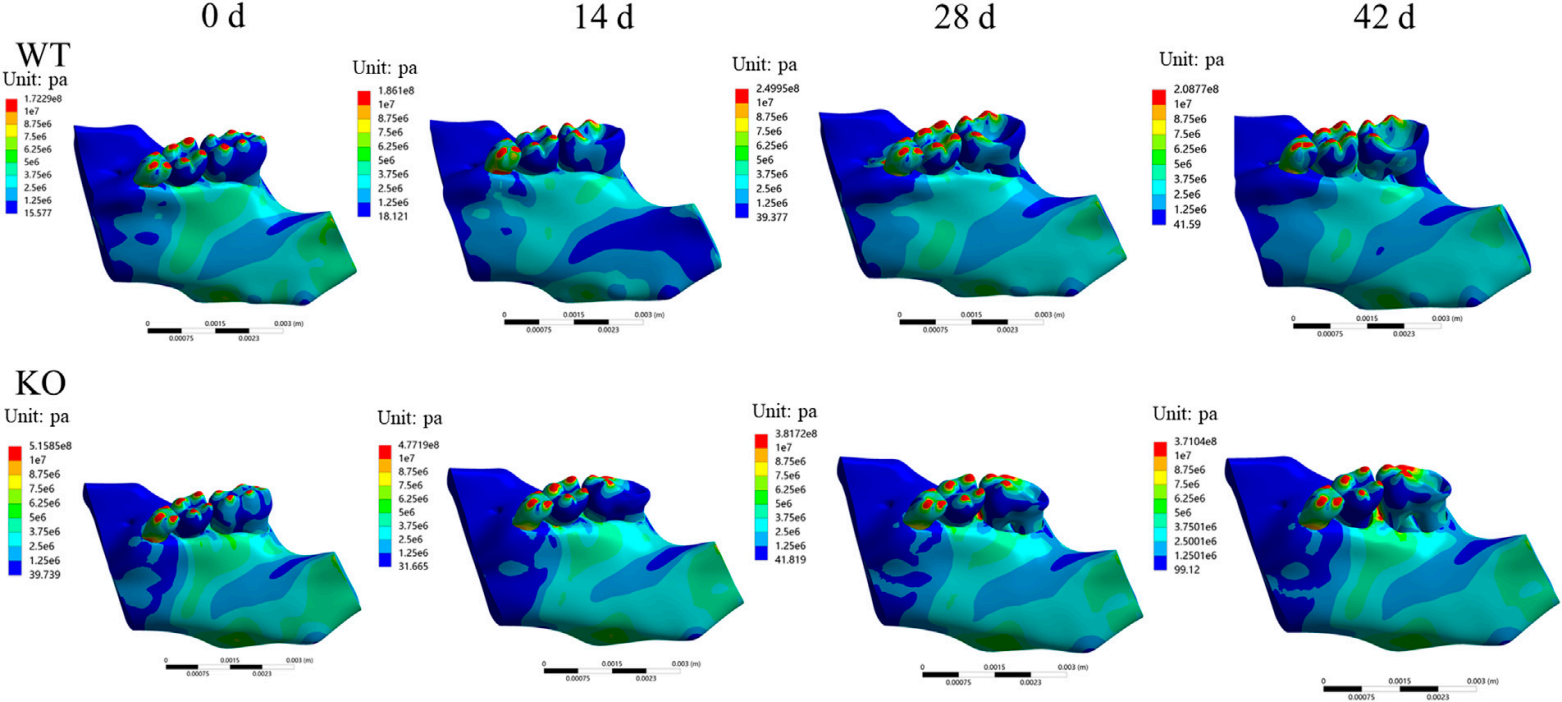
Three-dimensional reconstruction of periapical lesions in mice. Representative images of tooth in the KO group and WT group. Progressive periodontal bone loss and root exposure over time.

Micro-CT analysis was used to quantify the area of mineralized tissue and the apical lesion area of each mouse (Table 2). The apical lesion area increased gradually over time, and the root furcation lesion area also increased gradually. In the KO group, root apices were open with larger apical lesions (Figure 3). However, the difference between the two groups was not statistically significant (*p* > 0.05). The 3D reconstruction showed that the bone defects of the tooth cervix in the KO and WT mice gradually increased, and the roots became exposed (Figure 4).

**TABLE 2:** Apical lesion of each mouse.

Periapical lesion size (mm^2^)	KO	WT	P
14 days (median)	0.0565	0.0425	0.0873
28 days (median)	0.115	0.094	0.5476
42 days (median)	0.1805	0.1695	0.9999

Notes: WT: wild type; KO: knockout.

### Maximum stress and strain distributions of the alveolar fossa in different stages

**FIGURE 5:**
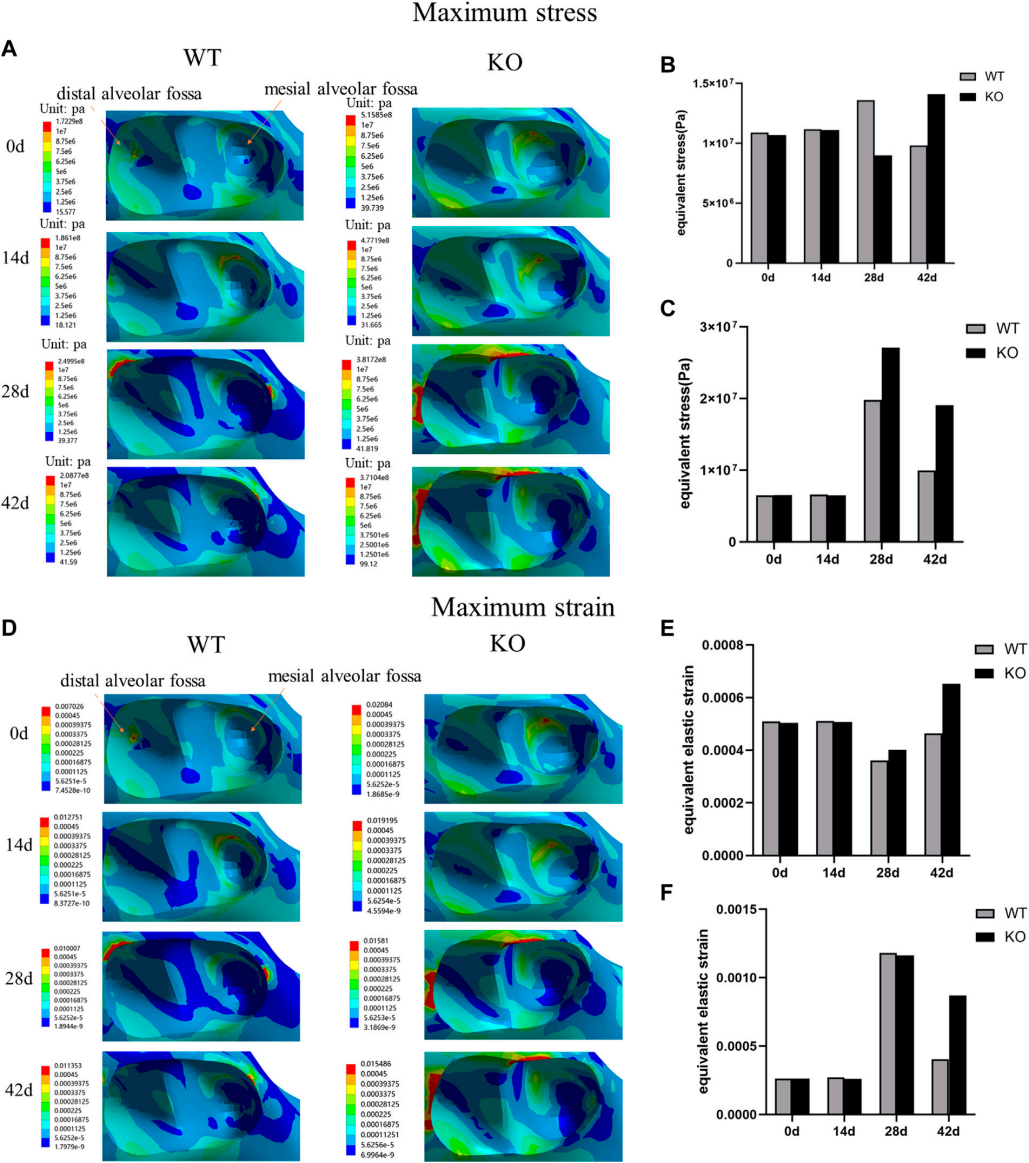
**(A)** Maximum stress of the alveolar bone of the left mandibular first molar in WT and KO mice under the vertical loading of 8 N·m. **(B)** Maximum stress of alveolar bone at tooth root. **(C)** Maximum stress of alveolar bone at tooth cervix. **(D)** Maximum strain of the alveolar bone of the left mandibular first molar in WT and KO mice under the vertical loading of 8 N·m. **(E)** Maximum strain of alveolar bone at tooth root. **(F)** Maximum strain of alveolar bone at tooth cervix.

**FIGURE 6:**
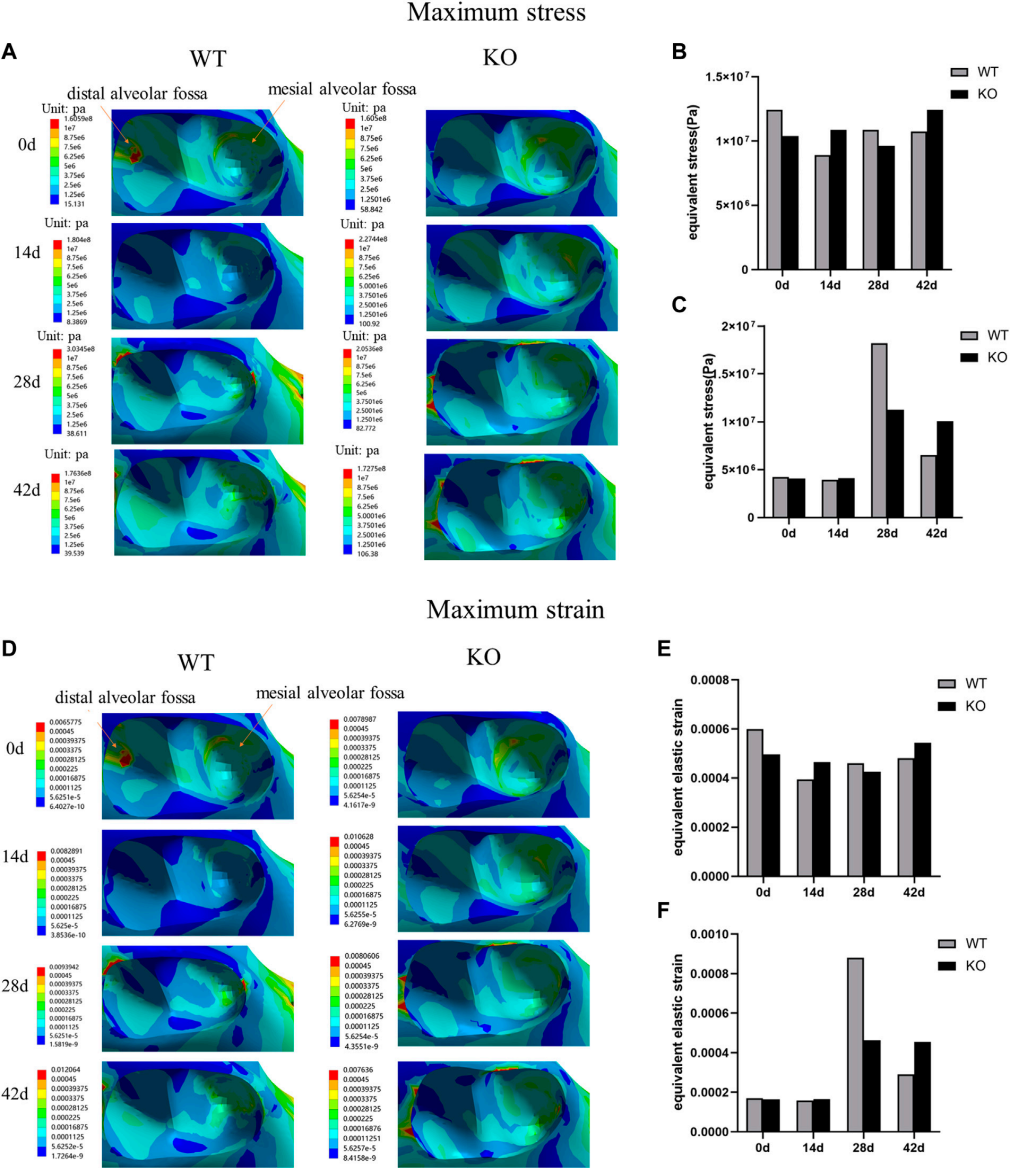
**(A)** Maximum stress of the alveolar bone of the left mandibular first molar in WT and KO mice under the buccal loading of 5 N·m. **(B)** Maximum stress of alveolar bone at tooth root. **(C)** Maximum stress of alveolar bone at tooth cervix. **(D)** Maximum strain of the alveolar bone of the left mandibular first molar in WT and KO mice under the buccal loading of 5 N·m. **(E)** Maximum strain of alveolar bone at tooth root. **(F)** Maximum strain of alveolar bone at tooth cervix.

**FIGURE 7:**
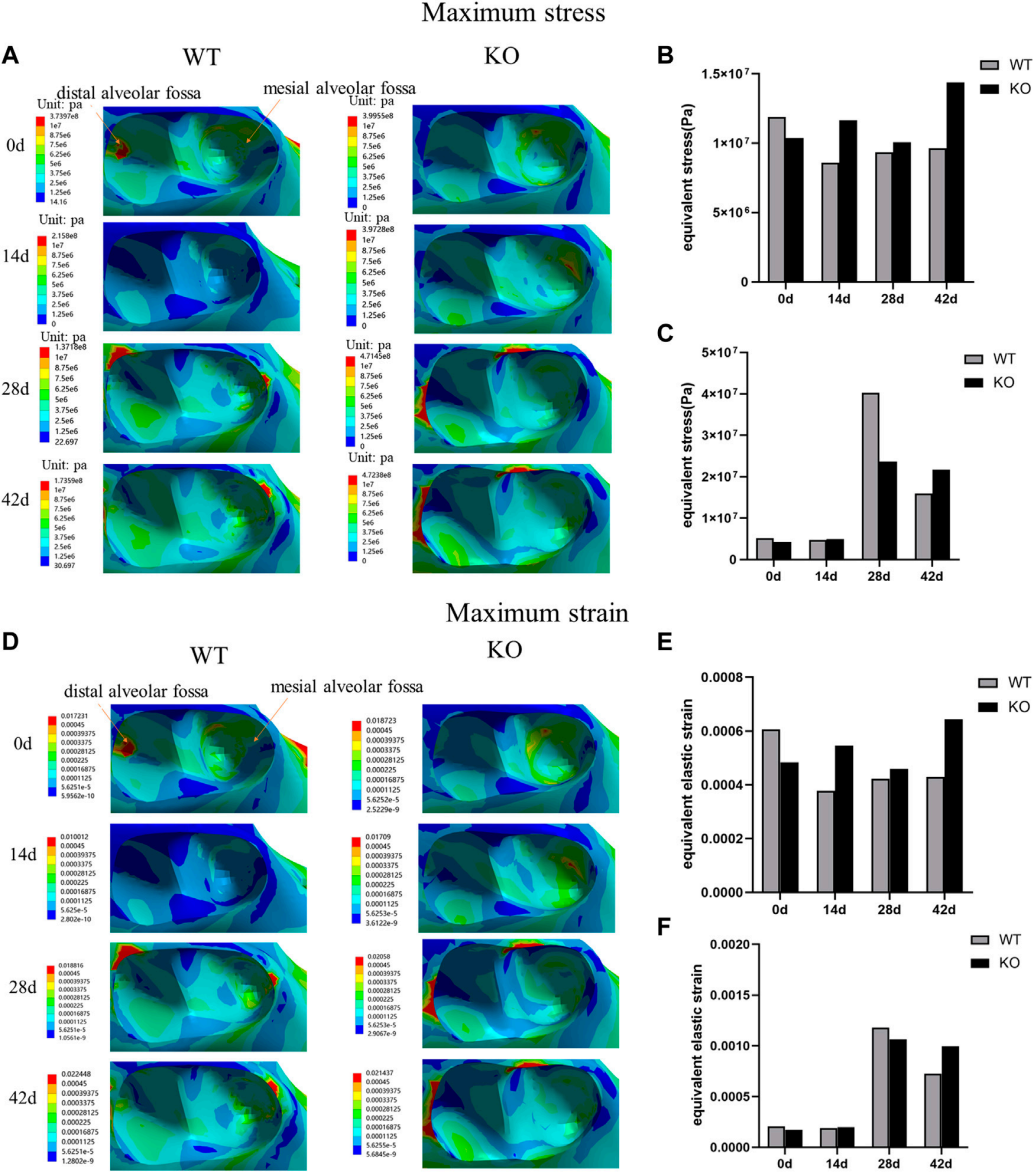
**(A)** Maximum stress of the alveolar bone of the left mandibular first molar in WT and KO under the lingual loading of 5 N·m. **(B)** Maximum stress of alveolar bone at tooth root. **(C)** Maximum stress of alveolar bone at tooth cervix. **(D)** Maximum strain of the alveolar bone of the left mandibular first molar in WT and KO mice under the lingual loading of 5 N·m. **(E)** Maximum strain of alveolar bone at tooth root. **(F)** Maximum strain of alveolar bone at tooth cervix.

In both WT and KO mice, the maximum stress and strain of the dentoalveolar bone were concentrated on the alveolar fossa at the tooth root at days 0 and 14. The maximum stress and strain were concentrated on the alveolar fossa at the tooth cervix at days 28 and 42 (Figures 5–7). When the tooth was in vertical and lateral occlusal contact, the stress in the alveolar fossa at the tooth cervix gradually increased from days 0–28 and decreased at days 42. At 42 days, the maximum stress and strain in KO mice were greater than those in WT mice.

### Effects of BMP9 on the maximum stress and strain of the alveolar fossa

**FIGURE 8:**
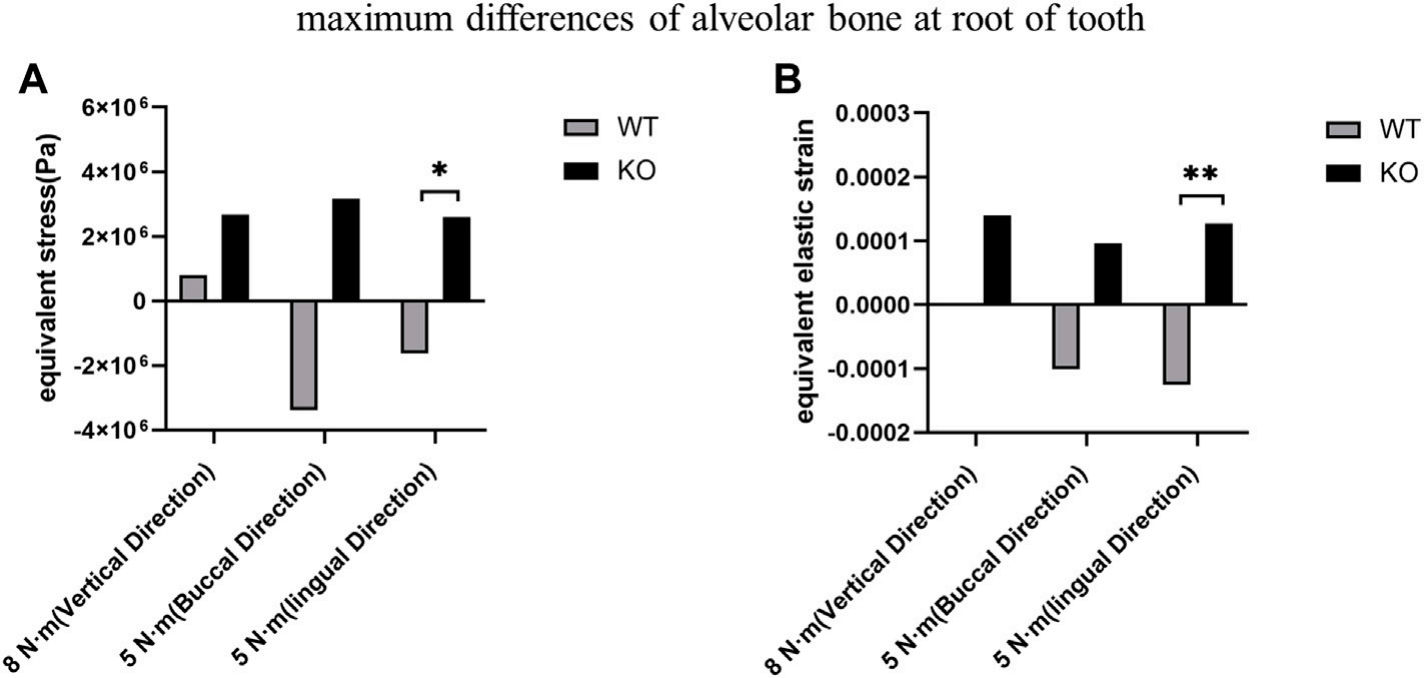
Comparison of the maximum differences between KO mice and WT mice. **(A)** Maximum stress differences of the alveolar bone at the tooth root. **(B)** Maximum strain differences of the alveolar bone at the tooth root.

The differences of maximum stress and strain of KO mice were greater than those of WT mice in three directions (Table 3). The differences of maximum stress and strain at the tooth root of KO mice were significantly higher than those of WT mice at lingual loading (*p* < 0.05). There was no significant difference in the differences of maximum stress and strain at the tooth cervix between the KO and WT mice (*p* > 0.05) (Figure 8).

**TABLE 3:** Maximum changes in the alveolar bone at the tooth root.

Maximum changes	Loads	KO					WT				
		A	B	C	D	E	A	B	C	D	E
Stress	8 N·m^a^	9.00E+06	2.69E+06	6.87E+05	1.51E+07	−6.82E+05	4.38E+06	−1.63E+06	2.52E+06	−3.55E+06	8.08E+05
	5 N·m^b^	−6.70E+04	3.16E+06	3.00E+04	6.01E+06	3.75E+06	−3.38E+06	4.47E+06	−4.16E+06	−4.32E+06	3.20E+05
	5 N·m^c^	7.95E+06	1.72E+06	9.92E+05	1.37E+07	2.59E+06	1.07E+06	−3.22E+06	−5.00E+06	−1.62E+06	1.23E+06
Strain	8 N·m^a^	2.68E-04	1.40E-04	−6.46E-05	7.64E−04	−2.50E-05	1.51E-04	−7.09E-05	1.22E-04	−1.72E-04	3.21E-06
	5 N·m^b^	−1.14E-04	5.70E-05	9.55E-05	2.23E−04	1.03E–04	−1.69E-04	1.18E-04	−1.01E-04	−2.71E-04	2.76E-04
	5 N·m^c^	2.41E-04	1.27E-04	7.30E-05	6.06E-04	8.70E−05	3.63E-05	−1.26E-04	−2.30E-04	−2.68E-04	−8.20E-05

Notes: WT: wild type; KO: knockout; a: vertical direction; b: buccal direction; c: lingual direction.

### Hematoxylin and eosin and artrate-resistant acid phosphatase staining

**FIGURE 9:**
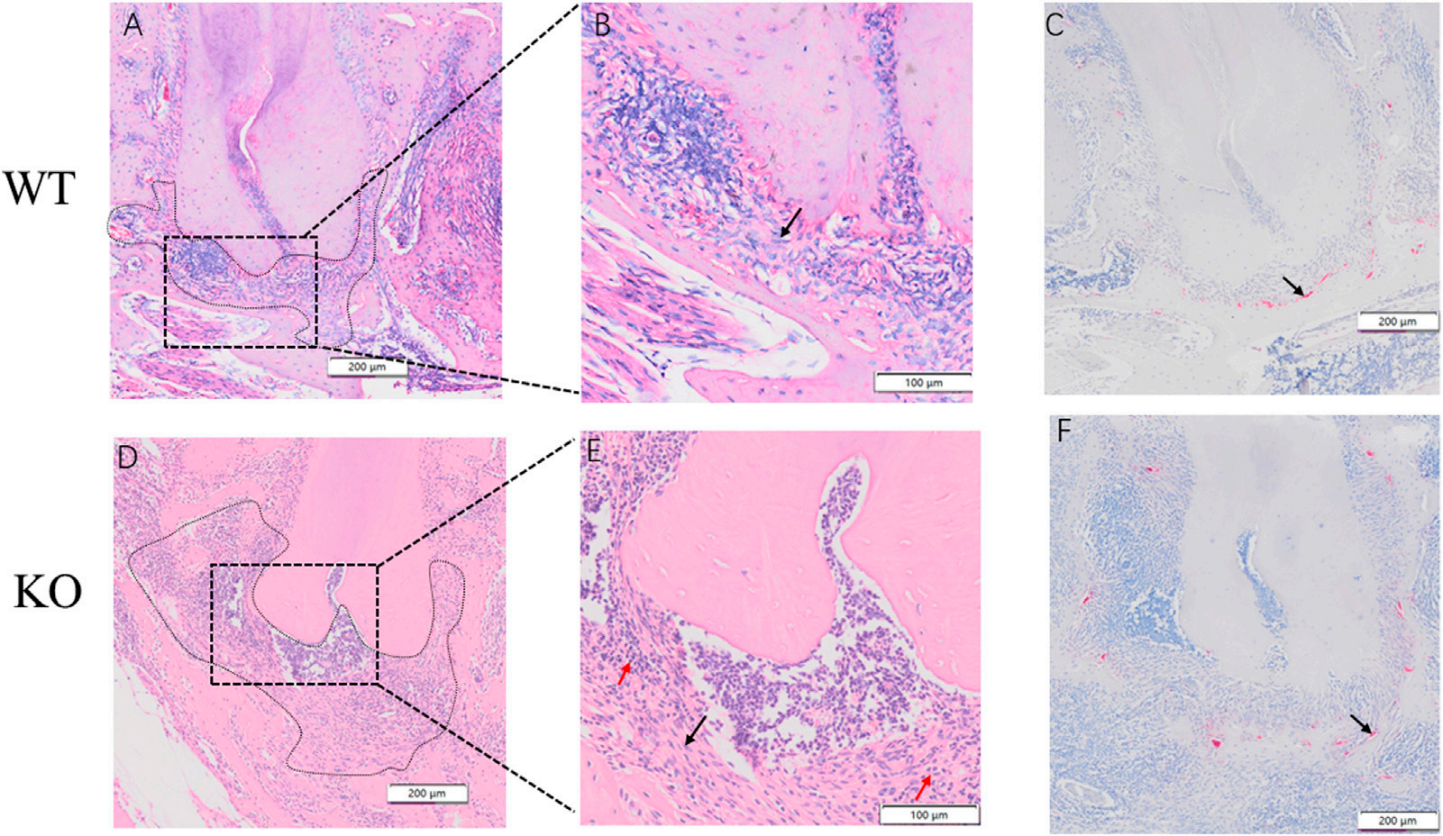
Hematoxylin and eosin **(H&E)** and Tartrate-resistant acid phosphatase (TRAP) staining of dentoalveolar bone in WT an KO mice. Periapical regions and fibroblasts (black arrow) of WT mice were observed by optical microscopes in **(A)** and **(B)**. Periapical regions, neutrophils (red arrow) and fibroblasts (black arrow) of KO mice were observed under optical microscopes in **(D)** and **(E)**. An osteoclastic cell can be observed in **(C)** an **(F)** (black arrow). Scale bars: 200 μm, 100 μm.

The periodontal ligament in the WT group was enlarged, showing dense inflammatory infiltration throughout the area (Figures 9A,B). In the KO group, the periodontal ligament was severely enlarged with fibrosis. Dense mixed inflammatory cell infiltration with abundant neutrophils was observed throughout the area in the KO group (Figures 9D,E). Osteoclasts were observed in both WT and KO groups (Figures 9C,F). Osteoclasts in the KO group were more active and had a wider distribution.

## Discussion

In this study, the biomechanics of the dentoalveolar bone during the development of apical periodontitis was investigated. Furthermore, we compared the biomechanics of the dentoalveolar bone between the WT mice and the KO mice with apical periodontitis. First, we found that the stress and strain at the alveolar fossa gradually concentrated on the tooth cervix, where the stress and strain increased with time but decreased at day 42. The maximum differences in stress and strain at the tooth root in the KO mice were greater than in the WT mice under the lingual loading. Therefore, BMP9 may affect the biomechanical response of dentoalveolar bone.

Under normal circumstances, the stress distribution of the tooth is mainly concentrated on the tooth root (Agrawal et al., 2021). When apical periodontitis occurs, the structure of the tooth root is gradually destroyed (Márton and Kiss, 2000; Xu et al., 2019; Luo et al., 2022), so the stress and strain on the tooth root may be reduced. In this study, we found the stress and strain at the tooth root to be significantly greater than at the tooth cervix at 0 and 14 days, which corresponds to the stress and strain distribution of the tooth under normal conditions. However, as the inflammation progressed, the stress and strain on the tooth cervix increased significantly at 28 days and the stress and strains on the tooth cervix with different loadings were all greater than those on the tooth root. These results indicate that the biomechanical homeostasis of the tooth is damaged during the development of apical periodontitis, especially in the tooth cervix, which may contribute to the slow destruction and disintegration of the structure of the tooth cervix.

As some studies found a significant correlation between apical periodontitis and marginal bone loss (Jansson et al., 1993; Jansson et al., 1995; Jansson and Ehnevid, 1998), our findings explain the potential impact of biomechanical changes on bone loss at the tooth cervix in apical periodontitis. In addition, the osteogenic and osteoclastic activities of dentoalveolar bone are in a highly dynamic equilibrium of bone homeostasis under normal conditions (Huang et al., 2020). The tooth with apical periodontitis may experience stress concentrations and therefore the tooth may be at risk of fracture (Petcu et al., 2013). This potential occlusal trauma could affect the healing of periapical disease (Harn et al., 2001). Our findings further explain the importance of controlling stress concentration in root canal therapy during the treatment of apical periodontitis. Therefore, in apical periodontitis, it is necessary to adjust the occlusion of the tooth to improve the abnormal biomechanical distribution of the tooth, thereby protecting the natural tooth and preventing further development of the disease.

Interestingly, we found that the stress and strain at the tooth cervix decreased at day 42. This may be due to the weakening of the bone-breaking effect and the gradual achievement of a new state of equilibrium (Nair, 2004). It is accepted that the immune response associated with apical periodontitis can be divided into an acute initial stage and a chronic stage (Stashenko et al., 1994). In the initial stage, pro-inflammatory cytokines are secreted, and bone resorption reaches a peak (Cavalla et al., 2021). In the chronic phase, pro-inflammatory cytokines are downregulated, reducing bone resorption, and the tissues reach equilibrium (Wang et al., 2011). However, this equilibrium can be disturbed if the inflammation continues to worsen. As inflammation progresses, the activities of osteoblasts and osteoclasts can reach a new equilibrium (Nair, 2004; Goldman et al., 2021). Therefore, the biomechanical properties of a tooth in apical periodontitis may be related to the activity of osteoclasts and osteoblasts.

In addition to studying the development of apical periodontitis in WT mice, we also studied the development of apical periodontitis in BMP9 KO mice to investigate the role of BMP9 in apical periodontitis. Emerging evidence indicates that BMP9, one of the most potent osteoinductive BMPs, may play a critical role in preventing inflammation, responding to mechanical stress, bone homeostasis, and osteo/odontogenic regeneration (Wang et al., 2014; Mostafa et al., 2019; Liu et al., 2020; Chen et al., 2021). We have previously demonstrated that mechanical stretch enhances BMP9-induced osteoblastic lineage specification in C3H10T1/2 mesenchymal stem cells (Song et al., 2018). However, the role of BMP9 in apical periodontitis was unknown. In this study, the dentoalveolar bone at the tooth root of KO mice was subjected to greater stress and strain than in WT mice. Under the same lingual loading, the stress and strain at the tooth root in KO mice were significantly greater than in WT mice. However, there were no statistically significant differences in the stress and strain on the dentoalveolar bone at the tooth cervix. This indicates that the effect of BMP9 on the biomechanics of the dentoalveolar bone is mainly at the tooth root. Therefore, BMP9 may be involved in stress stimulation during the development of apical periodontitis. Also, the effect of BMP9 on the biomechanics of the dentoalveolar bone may be site-dependent.

This study further found that the apical lesion areas on micro-CT images in the KO mice had an increasing trend compared to the WT mice. Furthermore, the H&E and TRAP staining results demonstrated that the severity of apical periodontitis at the tooth root in the KO mice was greater than in the WT mice. At 42 days, the H&E staining showed the KO mice had significantly wider periodontal space and more neutrophil infiltration compared to the WT mice. This result indicated that BMP9 knockout might aggravate the inflammatory response and indirectly indicated that BMP9 has some potential for anti-inflammatory responses. Although BMP9 is less studied in apical periodontitis, some studies of other diseases have found that BMP9 has anti-inflammatory and anti-fibrotic effects, preventing macrophages and neutrophils from entering the tissues (Chen et al., 2017; Hassanisaber et al., 2019). TRAP staining showed that osteoclasts in the apical region of the BMP9 KO mice were more active and broader than in the WT mice, suggesting that BMP9 knockout may aggravate osteoclast activity. A previous study found that BMP9 KO mice exhibited defects in the alveolar-bone complex, suggesting that BMP9 could regulate dentin formation and promote teeth development (Huang et al., 2019). Other studies have shown that the knockout of BMP9 can silence many key signaling pathways (Liu et al., 2020), which are likely to aggravate the severity of periapical lesions. Therefore, BMP9 may be related to the inflammatory response of apical periodontitis and more evidence needs to be gathered to reveal potential mechanisms.

The present study has some limitations that should be noted. First, all structures and materials simulated in this study were assumed to be homogeneous and isotropic; however, the material properties may vary in different regions. Second, comprehensive analysis of the stress distributions of tooth is complicated because one must consider the influences of tooth morphology, the number of roots and their configuration, and the dimensions and directions of loads. The complexity of actual occlusal forces cannot be completely simulated with numeric methods. Third, although the direction of mandible movement is multi-dimensional, our study only simplified the movement of mandible into three directions: vertical, buccal, and lingual. However, the occlusal process is more complicated. Finally, in the process of animal modeling, the range of defects induced by apical periodontitis in each group was not completely consistent, which may affect the accuracy of the results.

## Conclusion

To conclude, this study established a mouse model of apical periodontitis to study the effect of BMP9 and dentoalveolar bone biomechanics on the development of apical periodontitis. The results revealed that stress and strain at the alveolar fossa were gradually concentrated on the tooth cervix, and gradually increased with the progression of apical periodontitis. Further, BMP9 knockout was found to increase the stress and strain of the alveolar fossa at the root of the mandibular first molar when apical periodontitis mice were subjected to vertical and lateral occlusal forces under the same lingual loading. Taken together, these results strongly suggest that BMP9 and biomechanical properties of the dentoalveolar bone are closely correlated with the pathological process of apical periodontitis.

## Data Availability

The raw data supporting the conclusions of this article will be made available by the authors, without undue reservation.
